# Differentiating interstitial lung diseases from other respiratory diseases using electronic nose technology

**DOI:** 10.1186/s12931-023-02575-3

**Published:** 2023-11-06

**Authors:** Iris G. van der Sar, Marlies S. Wijsenbeek, Gert-Jan Braunstahl, Jason O. Loekabino, Anne-Marie C. Dingemans, Johannes C. C. M. In ‘t Veen, Catharina C. Moor

**Affiliations:** 1 Department of Respiratory Medicine, Center of Excellence for Interstitial Lung Disease, Erasmus University Medical Center, Rotterdam, The Netherlands; 2 Department of Respiratory Medicine, Erasmus MC Cancer Institute, University Medical Center, Rotterdam, The Netherlands; 3 Department of Respiratory Medicine, Franciscus Gasthuis & Vlietland, Center of Excellence for Asthma, COPD, and Respiratory Allergy, Rotterdam, The Netherlands

**Keywords:** Breath test, Diagnostic test, Biomarker, Electronic nose, Interstitial lung diseases, Obstructive lung disease, Lung cancer

## Abstract

**Introduction:**

Interstitial lung disease (ILD) may be difficult to distinguish from other respiratory diseases due to overlapping clinical presentation. Recognition of ILD is often late, causing delay which has been associated with worse clinical outcome. Electronic nose (eNose) sensor technology profiles volatile organic compounds in exhaled breath and has potential to detect ILD non-invasively. We assessed the accuracy of differentiating breath profiles of patients with ILD from patients with asthma, chronic obstructive pulmonary disease (COPD), and lung cancer using eNose technology.

**Methods:**

Patients with ILD, asthma, COPD, and lung cancer, regardless of stage or treatment, were included in a cross-sectional study in two hospitals. Exhaled breath was analysed using an eNose (SpiroNose) and clinical data were collected. Datasets were split in training and test sets for independent validation of the model. Data were analyzed with partial least squares discriminant and receiver operating characteristic analyses.

**Results:**

161 patients with ILD and 161 patients with asthma (n = 65), COPD (n = 50) or lung cancer (n = 46) were included. Breath profiles of patients with ILD differed from all other diseases with an area under the curve (AUC) of 0.99 (95% CI 0.97–1.00) in the test set. Moreover, breath profiles of patients with ILD could be accurately distinguished from the individual diseases with an AUC of 1.00 (95% CI 1.00–1.00) for asthma, AUC of 0.96 (95% CI 0.90–1.00) for COPD, and AUC of 0.98 (95% CI 0.94–1.00) for lung cancer in test sets. Results were similar after excluding patients who never smoked.

**Conclusions:**

Exhaled breath of patients with ILD can be distinguished accurately from patients with other respiratory diseases using eNose technology. eNose has high potential as an easily accessible point-of-care medical test for identification of ILD amongst patients with respiratory symptoms, and could possibly facilitate earlier referral and diagnosis of patients suspected of ILD.

**Supplementary Information:**

The online version contains supplementary material available at 10.1186/s12931-023-02575-3.

## Background

Worldwide, over 500 million people suffer from a respiratory disease and numbers are increasing, including numbers of patients with interstitial lung disease (ILD). However, ILDs still remain rare diagnoses. The overall global prevalence of ILD is approximately 0.09% [1]. Due to the lack of knowledge on ILD and the non-specific symptoms, recognizing patients suspected for ILD is poor amongst primary care physicians and community hospitals [2, 3]. Besides nonspecific disease presentation, various patient and healthcare related factors play a role [4]. Moreover, lung function is often still preserved in early ILD. A median delay of up to 2,1 years from start of symptoms until diagnosis has been reported and has been associated with worse outcomes [3, 5, 6]. Therefore, a non-invasive, less costly, accessible and reliable test to improve the diagnostic process is highly needed [7].

An electronic nose (eNose) device is a sensor-based technique that detects and profiles volatile organic compounds of exhaled breath non-invasively, without identification of the individual compounds. Both physiological and pathophysiological processes in the human body influence the volatile organic compounds; thus, exhaled breath provides valuable information about a person’s health.

Previous studies found that eNose technology can be used to accurately identify respiratory diseases, including ILD, lung cancer, asthma and chronic obstructive pulmonary disease (COPD) [8, 9]. In ILD, breath profiles of patients could be differentiated from healthy controls [10–14] and individual ILDs from COPD [11, 12]. Exploratory studies in pneumoconiosis show the potential of using an eNose for screening purposes in ILD [15, 16].

The aim of the current study is to investigate whether exhaled breath analysis using an eNose has potential as application for early detection of ILD amongst patients with respiratory symptoms. We assessed the accuracy of differentiating breath profiles of patients with ILD from patients with asthma, COPD, and lung cancer.

## Methods

### Study design

In this cross-sectional multicenter study patients were included at the outpatient clinic of the department of respiratory medicine of two hospitals in Rotterdam, the Netherlands: Erasmus University Medical Center (recognized expert center for ILD and lung cancer) and Franciscus Gasthuis & Vlietland (recognized expert center for asthma and COPD).

Patients with a diagnosis of ILD, asthma, COPD or lung cancer, regardless of stage or treatment were included in both hospitals between January 2019 and December 2022. ILD diagnosis was established by a multidisciplinary team according to the most recent guidelines [17–19]. At time of diagnosis, patients were diagnosed and classified for asthma following the applicable Global Initiative for Asthma guidelines [20], and for COPD following the Global initiative for chronic obstructive lung disease (GOLD) guidelines [21]. All patients with lung cancer had a pathology proven diagnosis. Patients with another lung disease, lung carcinoma in situ, current pulmonary infection or recent alcohol intake (< 8 h) were excluded.

### Data collection

The eNose used for exhaled breath analysis was the SpiroNose (Breathomix, Leiden, The Netherlands). This eNose contains seven different metaloxide semiconductor sensors in various arrays on both the inside and outside of the device [22, 23]. Each included patient performed one measurement that consisted of two breath maneuvers. One maneuver comprises five tidal breaths, an inhalation to total lung capacity, followed by a 5 s breath hold and a slow maximum expiration. Data were collected in an online platform that has a secured certified database (BreathBase). More details about the breath maneuver and breath data collection were described previously [23].

Participants completed a short questionnaire, including demographics, smoking history, and recent medication, food or drink intake. Other patient characteristics, medical history, medication use, and most recent available diagnostic test results (e.g., spirometry, chest imaging, pathologic assessment, blood samples) were collected from medical files.

### Data analysis

#### Pre-processing

Sensor data was extracted from the BreathBase platform and pre-processed before analysis. Pre-processing includes selection of the best breath maneuver, data correction for ambient air, data scaling to the most stable sensor, and reduction of inter-array differences [22, 23]. For each sensor, the peak value and the ratio between peak value and breath hold are used for statistical analyses. The peak value of the most stable sensor is excluded, resulting in 13 values per measurement (i.e. the breath profile) labeled with the collected patient characteristics. Measurements of insufficient quality caused for example by wrong breathing technique or unstable ambient air are removed.

#### Dataset and analysis groups

To answer the main aim of the study, breath profiles of patients with ILD were compared to the whole group of patients with another respiratory diagnosis (asthma, COPD or lung cancer). The four diagnosis groups were also compared separately. Moreover, patients with lung diseases that often have similar patient characteristics and risk factors (idiopathic pulmonary fibrosis (IPF), COPD, lung cancer) were compared.

A thorough power calculation was not possible, as data from previous similar studies were not available. We aimed to include enough patients in each diagnosis group to be able to split the groups in a training and test set in order to independently validate the results. Looking at eNose studies, a dataset size of ≥ 30 patients is generally sufficient to split [8]. To avoid imbalance between groups and reduction of statistical power of the model, larger groups were reduced by random patient selection using the function ‘sample’ in R [24].

To assess the influence of smoking on the accuracy of findings, comparison of breath profiles from patients with ILD versus all other diseases was repeated in patients who ever smoked. Moreover, the possible influence of medical center was assessed by comparing breath data of patients with asthma and COPD who were included in Erasmus Medical Center versus Franciscus Gasthuis & Vlietland. Lastly, breath data of all patients were compared based on their sex (males versus females) or smoking history (ever versus never, current versus former smokers) to test for the influence of these potential confounders.

Descriptive statistics were used to analyze baseline data, including χ^2^, Student’s t, and Mann Whitney tests to compare groups. We displayed normally distributed data as mean values (± standard deviation) and non-normally distributed data as median values (interquartile range). R version 4.2.1 for Windows with mixOmics version 6.20.0 package was used for analysis.

#### Data classification

The supervised classification technique partial least squares discriminant analysis (PLS-DA) was used to reduce dimensionality of breath profiles, and to classify and compare groups. Dimensionality reduction resulted in multiple principal components (PCs), which are weighted combinations of input variables (i.e. sensor values). If data was split in a training and test set, the first two PCs were used to assess the discriminative ability of eNose technology. If a dataset was not split, one PC was used to avoid model overfitting. Receiver operating characteristics analysis was applied to calculate the corresponding area under the curve (AUC) with 95% confidence interval (CI), sensitivity, specificity, accuracy, negative predictive value (NPV), and positive predictive value (PPV).

The presence of outliers in the values of PC1 and 2 were assessed. Outliers were defined as measurements outside upper and lower limits of a box-and-whisker plot. Limits were calculated as quartile 1 and 3 ± 1.5 * interquartile range. The main analysis was repeated without outliers to assess influence of outliers on the main results.

#### Probability score prediction of individual patients

To show the eNose performance in clinical practice based on our trained PLS-DA model, one hundred patients with ILD were randomly selected from the part of our dataset that was left out from previous analyses and not used for training or testing the model (i.e. unseen data). For each patient an individual probability score was calculated (range 0–1) using the ‘predict’ function in R. This function predicts how well the new patient data fit the average ILD breath profile that resulted from the trained PLS-DA model (i.e. PC1 and PC2). The higher the individual probability score, the better the breath profile of the patient fits the ILD breath profile. A density plot (i.e. relative likelihood against probability score*100%) was created to display the distribution of probability scores for all one hundred unseen dataset of patients with ILD.

## Results

### Baseline characteristics

**Fig. 1:**
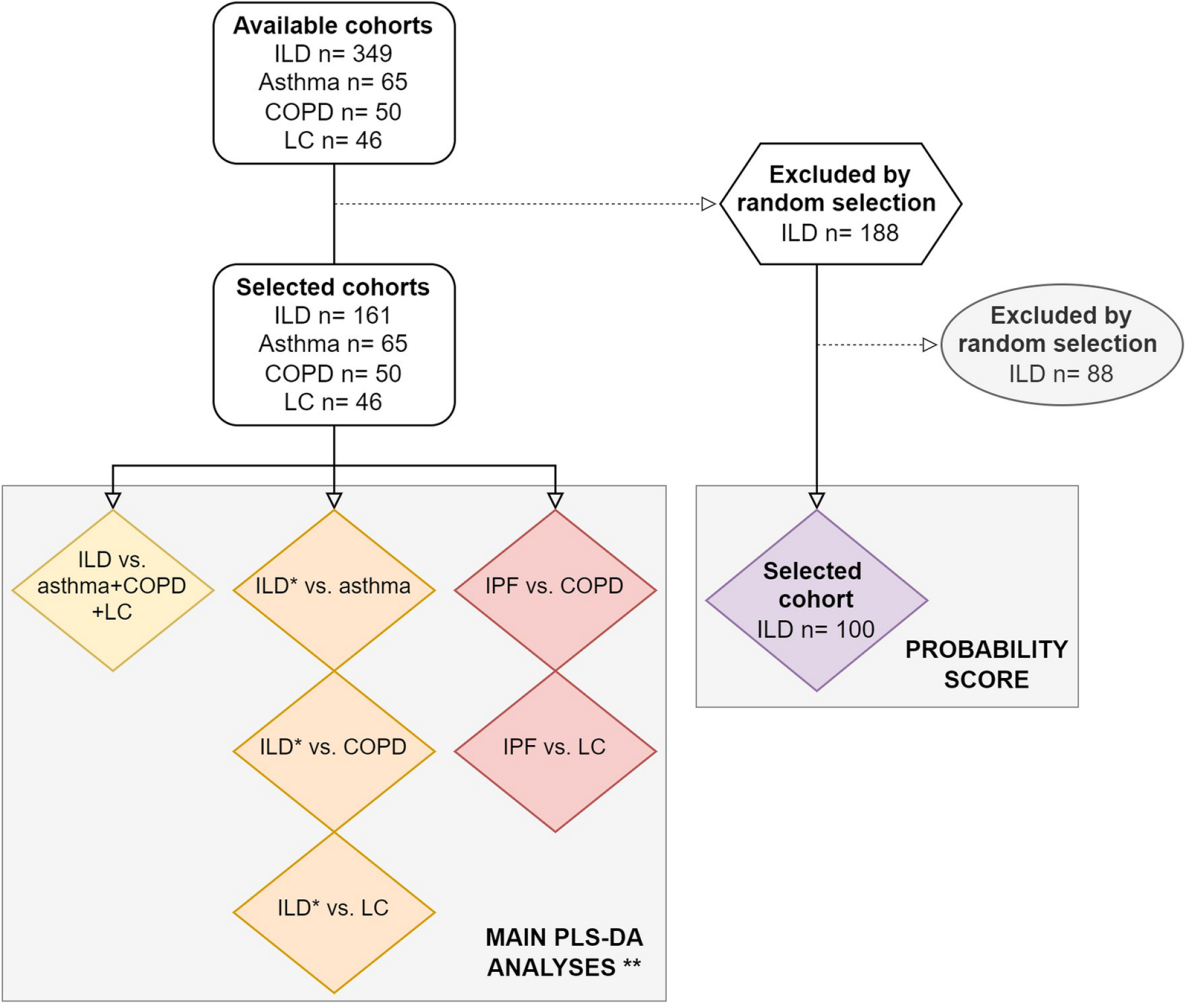
Flowchart of cohort selection for main analyses. Subgroup analyses are not included in this flowchart. IPF cohort existed of n = 61 patients. *Subgroups reduced to size n = 55 by random selection. **If group size ≥ 30, cohorts were split in a training and test set. COPD = chronic obstructive pulmonary disease; LC = lung cancer; ILD = interstitial lung disease; IPF = idiopathic pulmonary fibrosis; PLS-DA = partial least squares discriminant analysis

322 patients were included in this study; 161 patients with ILD were selected (from a total cohort of n = 349) to compare to 161 patients with other respiratory diseases (65 with asthma, 50 with COPD, and 46 with lung cancer). For comparing ILD with individual diagnoses, a subset of 55 randomly selected patients with ILD was used. Baseline characteristics of the overall cohort and individual diagnosis groups are shown in Table 1. An overview of the selected patient cohorts for the main analyses is shown in a flowchart (Fig. 1).

**Table 1:** Baseline characteristics

	Overall	ILD	Asthma	COPD	Lung cancer	p-value
Subjects (n)	322	161	65	50	46	
Females (n)	154 (47.8)	60 (37.3)	45 (69.2)	23 (46.0)	26 (56.5)	< 0.01
Age (years)	68 [58, 75]	71 [62, 76]	56 [42, 67]	66 [61, 74]	69 [63, 75]	< 0.01
Smoking amount ~ (py)	32.6 (31.5)*	25.8 (23.3)	16.3 (15.9)	49.6 (38.7)	41.9 (38.4)	< 0.01
Smoking status						< 0.01
Never	96 (30.7)	48 (30.4)	37 (56.9)	0 (0.0)	13 (28.3)	
Former	193 (59.9)	110 (68.3)	23 (35.4)	31 (66.0)	27 (58.7)	
Current	30 (9.3)	2 (1.2)	5 (7.7)	17 (34.0)	6 (13.0)	
FVC (%pred)	84.7 (20.6)**	80.7 (20.8)	93.2 (16.7)	84.2 (20.4)	94.2 (22.1)	< 0.01
FEV1 (%pred)	77.3 (22.4)**	81.7 (19.0)	81.6 (21.0)	54.5 (21.3)	85.3 (21.1)	< 0.01
DLCOc (%pred)		51.0 (16.2)^#^				
Diagnosis or Stage (n)		IPF 61 (37.9) HP 27 (16.8) CTD-ILD 27 (16.8) iNSIP 11 (6.8) CPFE 7 (4.3) COP 6 (3.7) Other ILD 22 (13.7)		GOLD I 16 (32.0) GOLD II 20 (40.0) GOLD III 7 (14.0) GOLD IV 7 (14.0)	SCLC 4 (8.7) NSCLC 42 (91.3) –––––––––––– Stage I 2 (4.3) Stage II 0 (0.0) Stage III 5 (10.9) Stage IV 39 (84.8)	
Eosinophil count (10^9^/L)			0.2 [0.1, 0.4]**			
Use of immunosuppressants (n)		55 (34.2)^	7 (10.8)	4 (8.0)	9 (19.6)	
Use of other disease-specific medication (n)		Antifibrotic 44 (27.3)	Biological 14 (21.5) ICS 59 (90.8)	ICS 30 (50.0)	Targeted 30 (65.2) CT and/or IT 10 (21.7)	

Values are displayed as number (%), mean ± SD, or median [interquartile range]. Subgroup ‘other ILD’ includes interstitial pneumonia with auto-immune features, desquamative interstitial pneumonia, vasculitis, unclassifiable ILD, asbestosis, respiratory bronchiolitis-ILD, drug induced ILD, sarcoidosis, granulomatous-lymphocytic ILD. If available, lung function values post-bronchodilator are displayed

COP = cryptogenic organizing pneumonia; COPD = chronic obstructive pulmonary disease; CPFE: combined pulmonary fibrosis and emphysema; CT: chemotherapy; CTD: connective tissue disease; DLCOc: diffusing capacity for carbon monoxide corrected for hemoglobin level; FEV1: forced expiratory volume in the first second; FVC: forced vital capacity; GOLD: Global Initiative for Chronic Obstructive Lung Disease; HP: hypersensitivity pneumonitis; ICS: inhaled corticosteroid; ILD: interstitial lung disease; iNSIP: idiopathic non-specific interstitial pneumonia; IPF: idiopathic pulmonary fibrosis; IT: immunotherapy; (N)SCLC: (non-)small cell lung cancer; py: pack years; %pred: percent of predicted value, calculated based on sex, age and height

~ never smokers (n = 99) excluded. * n = 7 missing values. ** n = 39 missing values. ^#^ n = 12 missing values. ^In case of prednisone: dosage ≥ 10 mg

### Main results

**Fig. 2:**
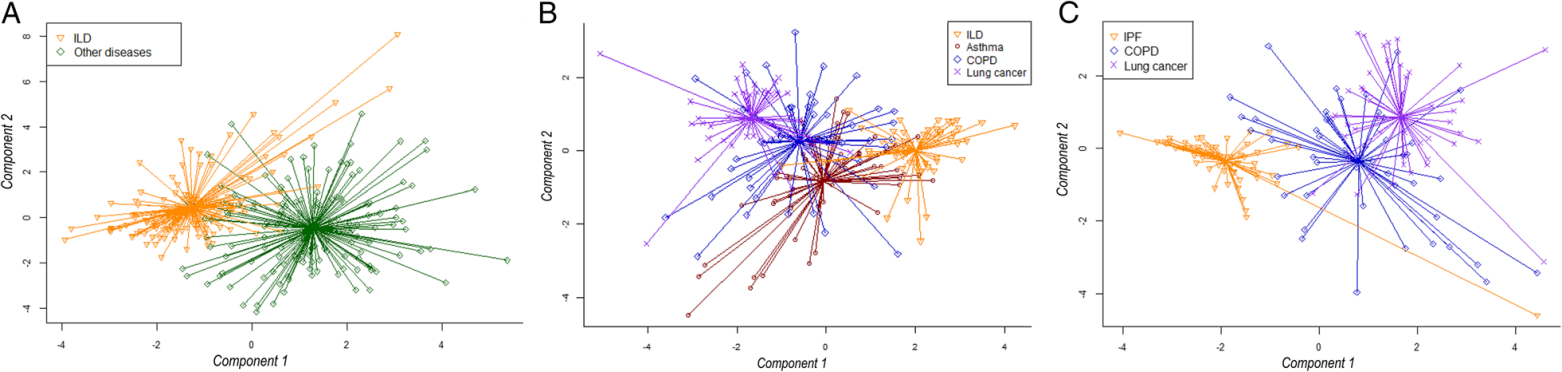
Comparison of breath profiles between patients with ILD and other respiratory diseases. **A** Scatterplot of patients with ILD (n = 161) versus other respiratory diagnoses (i.e. asthma, COPD, and lung cancer; n = 161). **B** Scatterplot of patients with ILD (n = 55) versus asthma (n = 65) versus COPD (n = 50) versus lung cancer (n = 46). **C** Scatterplot of patients with IPF (n = 61) versus COPD (n = 50) versus lung cancer (n = 46). Each dot represents one patient. Component 1 and 2 are principal components resulting from partial least squares discriminant analysis. COPD: chronic obstructive pulmonary disease; ILD: interstitial lung disease; IPF: idiopathic pulmonary fibrosis

Breath profiles of patients with ILD differed from all other respiratory diseases with an AUC of 0.97 (95% CI 0.95–0.99) in the training and 0.99 (95% CI 0.97–1.00) in the test set (Fig. 2A). Comparison of ILD with asthma (AUC 1.00, 95% CI 1.00–1.00), with COPD (AUC 0.96, 95% CI 0.90–1.00) and with lung cancer (AUC 0.98, 95% CI 0.94–1.00) showed similar results in the test sets. Additionally, breath profiles of patients with COPD and lung cancer (AUC 0.97, 95% CI 0.90–1.00) and COPD and asthma (AUC 0.90, 95% CI 0.79–1.00) could be distinguished with high accuracies. A scatter plot in Fig. 2B visualizes how breath profiles of all individual disease groups relate to each other.

Figure 2C shows the distribution of breath profiles of patients with IPF, COPD, and lung cancer. Comparing IPF with COPD resulted in an AUC of 0.93 (95% CI 0.86–1.00), and IPF with lung cancer in an AUC of 0.93 (95% CI 0.82–1.00) in the test sets. Corresponding specificity, sensitivity, accuracy, NPV and PPV of all group comparisons can be found in Table 2.

**Table 2:** Results of breath analysis between patient groups

Group 1	n =	Group 2	n =	Dataset	AUC	95% CI	Specificity	Sensitivity	Accuracy	NPV	PPV
ILD	108	Asthma–COPD–Lung cancer	108	Training	0.97	0.95–0.99	0.93	0.93	0.93	0.93	0.93
	53		53	Test	0.99	0.97–1.00	0.89	1.00	0.94	1.00	0.90
ILD	37	Asthma	44	Training	0.99	0.97–1.00	0.91	1.00	0.95	1.00	0.90
	18		21	Test	1.00	1.00–1.00	1.00	1.00	1.00	1.00	1.00
ILD	37	COPD	34	Training	0.97	0.97–1.00	1.00	0.86	0.93	0.87	1.00
	18		16	Test	0.96	0.90–1.00	0.94	0.89	0.91	0.88	0.91
ILD	37	Lung cancer	31	Training	1.00	1.00–1.00	1.00	1.00	1.00	1.00	1.00
	18		15	Test	0.98	0.94–1.00	0.89	1.00	0.94	1.00	0.88
COPD	34	Lung cancer	31	Training	0.88	0.79–0.97	0.88	0.87	0.88	0.88	0.87
	16		15	Test	0.97	0.90–1.00	1.00	0.93	0.97	0.94	1.00
COPD	34	Asthma	44	Training	0.92	0.85–0.98	0.95	0.76	0.87	0.84	0.93
	16		21	Test	0.90	0.79–1.00	0.86	0.88	0.86	0.90	0.82
IPF	41	COPD	34	Training	0.88	0.80–0.96	0.71	0.98	0.85	0.96	0.80
	20		16	Test	0.93	0.86–1.00	0.75	0.95	0.86	0.92	0.83
IPF	41	Lung cancer	31	Training	0.91	0.85–0.98	0.98	0.68	0.85	0.80	0.95
	20		15	Test	0.93	0.82–1.00	1.00	0.87	0.94	0.91	1.00

Results based on 2 principal components. AUC: area under the curve; CI: confidence interval; COPD: chronic obstructive pulmonary disease; ILD: interstitial lung disease; IPF: idiopathic pulmonary fibrosis; NPV: negative predictive value; PPV: positive predictive value

There were 25 outliers in the dataset. The outliers had no significant effect on the main results (see Additional file 1: Fig. S1 and Table S1).

### Predicted probability scores of individual patients

**Fig. 3:**
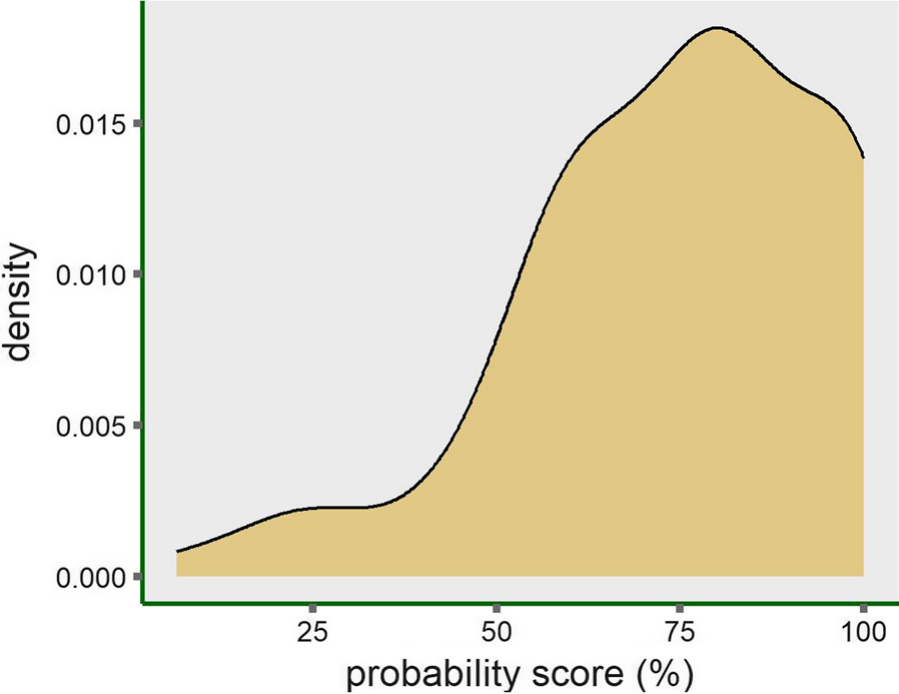
Predicted probability scores of individual patients with ILD based on breath data. Density plot shows the distribution of the predicted individual probability scores of a random sample of 100 unseen dataset of patients with ILD. Probability score is based on their breath profile and the trained PLS-DA model. The density (i.e. relative likelihood) is displayed on the y-axis and the individual probability scores on the x-axis

To illustrate how eNose might perform in future clinical practice, the probability of having an ILD was predicted based on eNose breath data of one hundred patients previously diagnosed with ILD. For example, a predicted probability of 88% means that the breath profile of this individual patient fits for 88% with the ILD breath profile. This might help physicians in clinical decision making. Figure 3 shows the distribution of all individual probability scores in a density plot.

### Subgroup results

An additional analysis on the influence of smoking is displayed in Table 3. eNose technology performed equally in the subgroup of ever smokers compared to the results of the full cohort. Moreover, breath profiles were not influenced by sex, or medical center. Current smokers seem to have slightly different breath profiles than former smokers.

**Table 3:** Results of breath analysis in subgroups

Group 1		Group2	n =	Dataset	AUC	95% CI	Specificity	Sensitivity	Accuracy	NPV	PPV
ILD (ever smoking)	75	Asthma–COPD–Lung cancer (ever smoking)	72	Training	0.99	0.99–1.00	0.99	0.96	0.97	0.96	0.99
	37		36	Test	0.94	0.89–0.99	0.86	0.95	0.90	0.94	0.88
Never smoker	96	Ever smoker	223		0.66	0.60–0.73					
Current smoker	30	Former smoker	193		0.80	0.73–0.87					
Male sex	168	Female sex	154		0.67	0.61–0.73					
Hospital EMC	254	Hospital FGV	73		0.64	0.53–0.74					

Results of the cohort that is split in a training and test set are based on 2 principal components; results of the unsplit cohort are based on 1 principal component. ^Includes asthma and chronic obstructive pulmonary disease patients only, as the number of patients with interstitial lung disease and lung cancer were too small in medical center FGV. AUC: area under the curve; CI: confidence interval; COPD: chronic obstructive pulmonary disease; EMC: Erasmus Medical Center; FGV: Franciscus Gasthuis & Vlietland; ILD: interstitial lung disease; NPV: negative predictive value; PPV: positive predictive value

## Discussion

Patients with ILD can be distinguished accurately from those with other respiratory diseases using eNose technology, shown in large training and test cohorts of patients with different disease stages and treatments. Moreover, the separation of breath profiles of patients with ILD compared to asthma, COPD or lung cancer individually was highly accurate, independent of age or sex. These results show the potential of using an eNose for detection of ILD non-invasively. If these findings are confirmed in a asymptomatic or early ILD patient cohort, screening or early detection might be possible.

Our results align with previously published results on the performance of eNose technology in differentiating ILD from COPD [11, 12]. Dragonieri et al. compared IPF with COPD and found an AUC of 0.85 in a test cohort, with active smokers being excluded [11]. The study of Krauss et al. aimed to differentiate individual ILDs, but patients with COPD were included as a control group [12]. Comparing CTD-ILD versus COPD resulted in an AUC of 0.85, and cryptogenic organizing pneumonia versus COPD in an AUC of 0.77. Other ILDs were not reported. Moreover, only patients with COPD GOLD stage III-IV were included, and results were not validated in a test set. Although direct comparison of results is difficult as both studies used another eNose device and selected patients with specific ILD diagnoses, all published results emphasize the potential of the overall concept of eNose technology for ILD. To our knowledge, studies that compare ILD with lung cancer or asthma have not been published until date.

No studies have been published on early detection of ILD using an eNose, except for two studies that focus on pneumoconiosis screening in high risk groups [15, 16]. Although these were pilot studies, they found high accuracies when comparing people with and without pneumoconiosis. Recently, studies on lung cancer screening have become available. A prospective study in patients with COPD showed that patients that developed lung cancer had a different breath profile already two years before the diagnosis of lung cancer compared to patients that did not develop lung cancer [25]. Moreover, De Kort et al. published a validation study on the performance of eNose technology for lung cancer screening [26]. They included patients suspected of lung cancer prior to tissue biopsy. In this robust study, the presence of lung cancer could be predicted using an eNose with an AUC of 0.79 in the validation cohort. This performance increased to an AUC 0.86 when known clinical risk factors where added in the model. These studies illustrate the promise of incorporating eNose results in risk models for early detection of respiratory diseases.

Interestingly, in our study we also found an accurate separation between patients in different clinically heterogenous subgroups with smoking-related diagnoses (IPF, COPD and lung cancer). The diagnostic workup of patients with unexplained respiratory symptoms and differentiation between various diagnoses is complex, especially in patients with similar clinical characteristics. Moreover, pulmonary function tests often do not show abnormalities in early disease. Thus, we believe that eNose technology could be of added value to raise early suspicion for ILD and improve referral and adequate diagnosis in both primary and secondary care.

Several limitations of our study should be named. First, we chose only one classification algorithm for data analysis. PLS-DA is an accepted method for classification of groups, but several methods should be compared in validation studies [27, 28]. Second, our study lacks an external validation cohort. We minimized the risk for model overfitting by splitting our dataset in a separate training and test set, but an external cohort is necessary to confirm the model performance. Besides, in our study cohort, the prevalence of ILD is much higher than would be expected in a real-life cohort of patients with unexplained respiratory symptoms. In a real-world setting, negative predictive value for ILD would therefore likely be higher, and positive predictive values lower. Lastly, the included cohort might not be representative for the overall population for which a clinical test for early disease detection is most beneficial; i.e. the patients visiting a physician with new or unexplained respiratory symptoms. The majority of the study cohort consisted of prevalent patients, of whom many already used disease-modifying treatment and had advanced disease stage. However, eNose technology achieved high accuracies despite the cohort heterogeneity in terms of treatment, stage and disease severity, indicating the suitability for application in real-world populations. Nevertheless, we should include patients with suspected and early respiratory diseases from primary health care centers and community sites in future multicenter external validation studies.

## Conclusion

eNose technology can be used to distinguish patients with ILD from patients with other respiratory diseases. This technology has high potential as an easily accessible point-of-care medical test for accurate identification of patients with ILD, and could facilitate earlier diagnosis and referral of patients suspected of ILD.

### Supplementary Information

**Additional file 1:** Outlier analysis.

## Data Availability

The datasets used and/or analysed during the current study are available from the corresponding author on reasonable request.

## Abbreviations

AUC: Area under the curve
CI: Confidence interval
COPD: Chronic obstructive pulmonary disease
eNose: Electronic nose
GOLD: Global initiative for chronic obstructive lung disease
ILD: Interstitial lung disease
IPF: Idiopathic pulmonary fibrosis
NPV: Negative predictive value
PC: Principal component
PLS-DA: Partial least squares discriminant analysis
PPV: Positive predictive value
