# Supplementary material for: Differentiating interstitial lung diseases from other respiratory diseases using electronic nose technology
Source: Respir Res. 2023 Nov 6;24:271. doi: 10.1186/s12931-023-02575-3 (PMC10626662; doi:10.1186/s12931-023-02575-3)
Supplement: Supplementary file 1 — Additional file 1: Outlier analysis. [file 12931_2023_2575_MOESM1_ESM.pdf]

Additional file 1

Outlier analysis

Figure S1: *Box-and-whisker plot of the first principal component resulting from breath profile comparison of patients with ILD and other chronic respiratory diagnosis.*

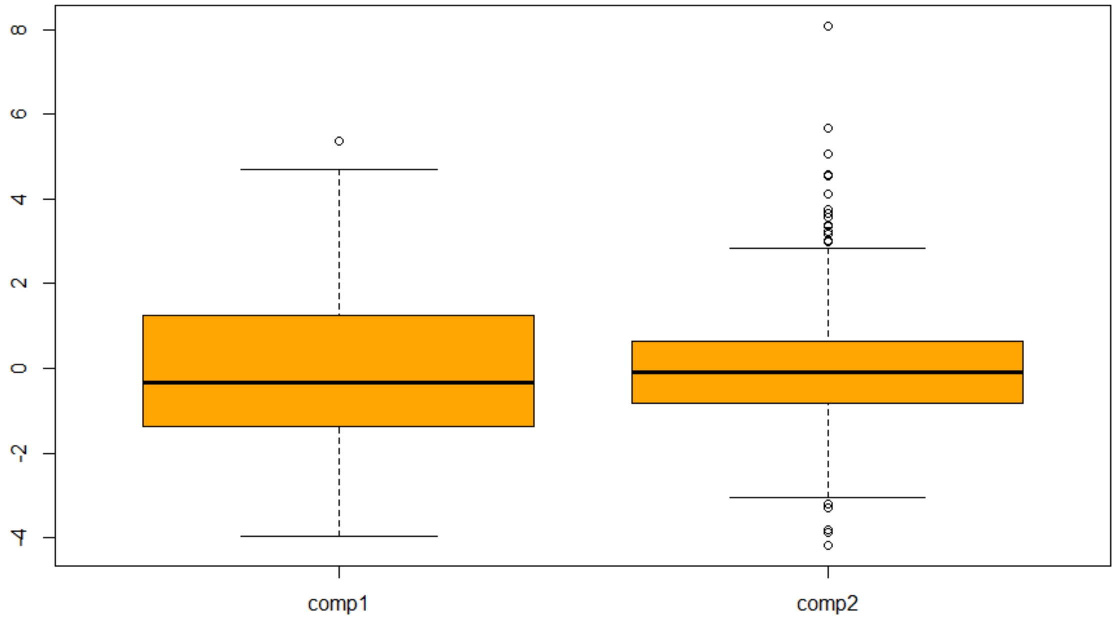

Principal component 1 (comp1) and 2 (comp2) result from the partial least squares analysis of breath profiles comparison of patients with ILD and other respiratory diseases. Outliers are marked as dots (n=25).

Table S1: *Results of breath analysis comparison without outliers.*

**Group 1**	n=	**Group 2**	n=	***Dataset***	***AUC***	*95% CI*	***Specificity***	***Sensitivity***	***Accuracy***	***NPV***	***PPV***
**ILD**	101	**Asthma – COPD – Lung cancer**	98	Training	0.97	*0.95-0.99*	0.91	0.95	0.93	0.95	0.91
	50		48	Test	0.98	*0.96-1.00*	0.94	0.94	0.94	0.94	0.94

Results based on 2 principal components. AUC = area under the curve; CI = confidence interval; COPD = chronic obstructive pulmonary disease; ILD = interstitial lung disease.
